# Quantum Walks in Periodic and Quasiperiodic Fibonacci Fibers

**DOI:** 10.1038/s41598-020-64065-6

**Published:** 2020-04-28

**Authors:** Dan T. Nguyen, Thien An Nguyen, Rostislav Khrapko, Daniel A. Nolan, Nicholas F. Borrelli

**Affiliations:** Science and Technology Division, Corning Research and Development Corporation, Sullivan Park, Corning, NY 14831 USA

**Keywords:** Quantum optics, Single photons and quantum effects

## Abstract

Quantum walk is a key operation in quantum computing, simulation, communication and information. Here, we report for the first time the demonstration of quantum walks and localized quantum walks in a new type of optical fibers having a ring of cores constructed with both periodic and quasiperiodic Fibonacci sequences, respectively. Good agreement between theoretical and experimental results has been achieved. The new multicore ring fibers provide a new platform for experiments of quantum effects in low-loss optical fibers which is critical for scalability of real applications with large-size problems. Furthermore, our new quasiperiodic Fibonacci multicore ring fibers provide a new class of quasiperiodic photonics lattices possessing both on- and off-diagonal deterministic disorders for realizing localized quantum walks deterministically. The proposed Fibonacci fibers are simple and straightforward to fabricate and have a rich set of properties that are of potential use for quantum applications. Our simulation and experimental results show that, in contrast with randomly disordered structures, localized quantum walks in new proposed quasiperiodic photonics lattices are highly controllable due to the deterministic disordered nature of quasiperiodic systems.

## Introduction

For the last decade there has been significant of efforts in the investigation of quantum walks (QWs) in photonics lattices or arrays of waveguides^1–5^. From fundamental research in the 2000s, QWs have become one of the key operations in a number of quantum protocols in quantum communication and information as well as in quantum computing and simulation. Quantum walks have been modeled for exponential speedup in quantum algorithms^6,7^, to implement universal quantum gates for quantum computers^8,9^ and quantum simulations^10,11^. Successful implementation of these protocols will have an important impact on the research and development of quantum technologies. The first scalable quantum simulations of molecular energies have been demonstrated a few years ago^12–14^ indicating that quantum revolution 2.0 may be viable and not very far away. Photons, the quanta of electromagnetic field are considered as excellent “walkers” as their generation and manipulation are highly controllable. Furthermore, photonics technology is very mature, and many photonics systems can be operated ideally in room-temperature conditions. That is a huge advantage of photonics as compared to other technologies used in quantum applications such as ion-trapped, superconducting operating at extremely low temperatures, often at tens of millikelvin (mK). In light of that direction, quantum simulations with light^15^ and photonics quantum gates^16^ have been proposed and developed recently. Quantum walks of photons can be realized in the form of discrete-time QWs using beam splitters^17,18^ or continuous-time QWs using evanescently coupled waveguides arrays or photonics lattices^1–5^.

Over the last ten years, localized quantum walks (LQWs) of photons has attracted a lot of attention and effort of investigation. Originally, the phenomenon of localization of quantum particles, the electrons in the presence of a disordered medium was theoretically predicted by Anderson in his paper published in 1958^19^. The phenomenon, later known as Anderson localization, is a result of destructive interreference of all scattering paths of a wave or a quantum particle propagating in a disordered medium. The effect of a randomly disordered medium to the wave/quantum particle evolution can be simulated by spatially and/or temporally randomizing operations of the system dynamics. By doing that, Anderson localization can be realized^20–25^. Since the first demonstrations of LQWs of photons in photonics lattices^20,21^, the phenomenon of LQWs has attracted great interests and efforts of research not only for fundamental understanding but also for applications. For example, there have been proposals of employing LQWs for secure transmission of quantum information^23^ and secure quantum memory^24^.

Recent discoveries in the field of localization of light are not only important to understand better Anderson localization, but can also be explored for important applications^25–29^. Meanwhile, it is well established that not only randomly breaking periodicity but deterministic deviations from the periodicity can also result in higher complexity leading to new and surprising effects. The very well-known examples of such deviated structures are photonic quasi-crystals, a class of structures made from basics elements that are arranged in patterns but lack translational or rotational symmetries. From that definition, a quasiperiodic structure can be considered as in between the randomly disorder systems and the periodic ones. Localization of light has been discovered as earlier as in 1980s in several photonic quasi-crystals^30,31^. In the following decades, the effect has been realized in other quasiperiodic structures such as quasi-crystalline Fibonacci dielectric multilayers (FDML)^32,33^, in semiconductor multiple quantum-wells^34^, two-dimensional (2D), and three-dimensional (3D) quasi-crystal structures^35,36^.

At this point, we want to stress that due to the randomness nature of Anderson localization, quantifying the localization effect would require great efforts. Usually, it requires a large number of realizations on many structures that have the same degree of disorder. The final results are averaging over a large number of realizations. For the same reasons, LQWs in photonics lattices have been conventionally realized on many arrays composed with evanescently coupled waveguides whose positions are randomly disordered. As a result, quantification of LQWs in disordered arrays of waveguides requires many such structures which is difficult in practice. More complicatedly, the randomness of those structures must be controlled within a defined range of the disorder so that the disorder-induced localization can be quantified^20–22^. From the description, experimental realizations of LQWs require great efforts and resources^20–22^. Meanwhile, it is well established that localization of light can be realized in quasi-crystals or quasiperiodic photonics structures^30–33^. In such cases, deviations from periodicity provide deterministic disorders lead to localizations in the quasiperiodic systems. Because of the nature of deterministic disorder in quasiperiodic structures, localization can be realized deterministically without averaging over many realizations. In that spirit, we have recently proposed theoretically new quasiperiodic arrays of waveguides that are constructed with quasiperiodic Fibonacci and other sequences^37,38^. These arrays of waveguides represent a new class of quasiperiodic photonics lattices (QPLs) possessing both on- and off-diagonal deterministic disorders that can be used for deterministically realizing LQWs^37,38^. Furthermore, the simple construction rules in the new proposed QPL make it easy to create symmetrically distributed QPLs. It is worth mentioning that, LQWs with symmetrical distributions are required for quantum memory applications^24^.

So far, QWs of photons have been investigated in a number of integrated photonics lattices, such as silicon oxynitride waveguides^1^, e-beam lithography fabricated waveguide lattices on an AlGaAs substrate^2,3^, laser-writing silica glass waveguides^4^ and borosilicate glass waveguides^5^. Note that, in these photonics lattices the waveguides in the ‘walking region’ are close enough to ensure evanescent couplings among them. It is well-known that, couplings between silicon-based waveguides (mode field diameter MFD ~ 1 micron) with single-mode (SM) optical fiber (typically MFD ~ 10 micron) are usually of very high loss (more than 20 dB) and require significant efforts. Moreover, intrinsic background losses in silicon waveguides (0.1 dB/cm to ~1 dB/cm) are few orders of magnitudes higher than that of optical fibers (~0.2 dB/km) for telecom signals. High insertion loss including intrinsic and coupling losses could be a very negative factor for scaling up numbers of waveguides in silicon-photonics lattices for realizing QWs. Meanwhile, laser-writing technique can write large number of waveguides on glass, for example a lattice of 49 × 49 = 2401 waveguides^5^. However, laser-written glass waveguides usually have small index contrasts, resulting in weak confinement and sensitivity to the imperfections and perturbations as clearly indicated in recent experiments in which QWs characteristics were completely distorted in just about 1 cm of propagation^5^. The optical fiber-based QWs systems such as multicore fibers would significantly reduce or even eliminate these problems. The multicore fibers with extremely low insertion loss would be critical for scalable platforms for QWs, and quantum photonics computing and simulations to solve large-size problems which are intractable on classical computing.

In this paper, we have demonstrated for the first time QWs and LQWs in a new type of optical fibers having cores constructed with periodic and quasiperiodic Fibonacci sequence, respectively. Good agreement between theoretical and experimental results has been achieved. The new multicore-ring fibers (MCRF) provide a new scalable platform for experiments of QWs and other quantum effects that require large numbers of waveguides. More importantly, our new Fibonacci MCRF (FMCRF) provide a new class of photonics lattices possessing both *on- and off-diagonal deterministic disorder* for realizing LQWs deterministically. The proposed FMCRFs are simple and straightforward to make and have a rich set of properties that are of potential use for quantum applications. Furthermore, our simulation and experiment results show that, in contrast with randomly disordered structures, LQWs in quasi-periodic ones are highly controllable due to the deterministic disordered nature of quasi-periodic systems. At this point we want to stress that our results, both modeling and experiment in this work are restricted to the problem of single-photon QWs in MCRFs. The phenomena, although we do not explore purely quantum or non-classical nature of photons, have played meaningful roles for understanding and developing protocols of quantum applications. More details of this point will be elaborated later in the Discussion and the Method sections of this paper.

## New Periodic and Quasiperiodic Multicore Ring Fibres

**Figure 1:**
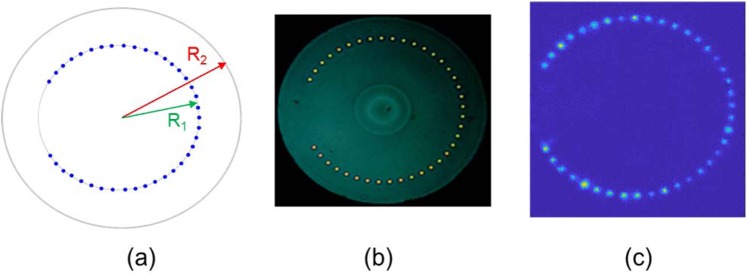
Diagram of MCRF with 39 identical SM cores (**a**), image of the fabricated fiber (**b**), and the image of the fiber that is overfilled with light of wavelength 1.550 μm.

In this section, we will present in details the concept of new multicore ring fibers as platform for realizing QWs and LQWs. In general, our new type of fibers - the multicore ring fibers (MCRF) are designed and fabricated with a configuration of a ring of cores and is compact in comparison with planar arrays of waveguides used to realize QWs on a line^1–4^. Figure 1 shows the diagram of MCRF and the image of the fabricated fiber of 39 cores and the image of fiber that is illuminated and overfilled with light of wavelength 1.55 μm. All the cores of the fiber have diameter *a* = 4.5 μm, *R*_1_ = 120 μm, and *R*_2_ = 160 μm.

The fiber shown in Fig. 1 is designed and fabricated with all cores of identical SM waveguides that are regularly *periodic* in a circular ring. The image of the overfilled fiber shows there is variation of the cores, and our characterizations show core diameter variation of about +/−2%. This periodic multicore ring fiber is called multicore-ring fiber (MCRF) for short. In our experiments of single-photon QWs, we will launch signal into the center core of the core ring, and the QWs process will take place from the center to the end cores of the two symmetrical arms. Notice that, the two end-cores of the two arms should not be too close to avoid coupling between these two cores that would distort distribution of the QWs on a line, which do not have such coupling in a planar array of waveguides^1–4^.

For the quasi-periodic multi-core ring fibers, the ring of cores is constructed with a Fibonacci sequence with two different SM waveguides *A* and *B* (the fiber is called Fibonacci MCRF or FMCRF). For example, waveguide *A* and *B* is characterized by *V*_A_ = π*a*_A_*NA*_A_/λ, and *V*_B_ = π*a*_B_*NA*_B_/λ, respectively, where *a*_A(B)_ stands for core diameter and *NA*_A(B)_ is the numerical aperture of waveguide *A*(*B*). Note that, the numerical aperture NA can be determined by the index difference between core and clad Δ*n* = *n*_core_ − *n*_clad_, and we will use Δ*n* to characterize waveguides in our calculations. In general, the construction rule for the cores of a fiber with Fibonacci j-th order are the same as in the Fibonacci arrays of waveguides or photonic lattices^37,38^. The difference between the two structures is the ring of cores in FMCRF instead of linear arrays of cores in Fibonacci array of waveguides. The construction rule for the cores of Fibonacci jth order is defined as

1 $${F}_{j}={S}_{j}{S}_{j-1}\ldots {S}_{2}{S}_{1}{S}_{2}\ldots {S}_{j-1}{S}_{j},$$

where *S*_*1*_*, S*_*2*_ *… S*_*j*_ are Fibonacci elements defined as

2 $${S}_{j}={S}_{j-2}{S}_{j-1},\,with\,{S}_{1}=A,\,{S}_{2}=B.$$

Here, *A* and *B* are two different SM waveguides as described above (see more details of the Fibonacci quasiperiodic photonic lattice in our previous works^37,38^).

**Figure 2:**
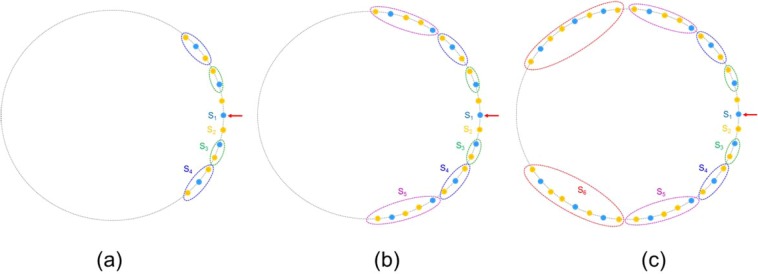
Diagram of ring of cores: 4^th^ order FCRF4 with 13 cores (**a**), 5^th^ order FCRF5 with 23 cores (**b**), and 6^th^ order FCRF6 fiber with 39 cores (**c**). Red arrow indicates input core.

As examples, Fig. 2 shows diagrams of structures of core rings of 4^th^, 5^th^ and 6^th^ order of FMCRFs. Notice that, these three core rings have the same ring radius for convenient discussion but not necessary. The red arrows indicate the input core, which is the center of the ring composed of two symmetrical arms. *S*_1_, *S*_2_ … *Sj* are the Fibonacci elements of *j*^th^ order defined in Eq. (2) above and are indicated by color circles. From the definition in Eq. (1), it is easy to calculate the number of cores for the 4^th^, 5^th^ and 6^th^ order FMCRFs are 13, 23 and 39, respectively. It is clear from Fig. 2 that the core rings constructed with Fibonacci sequences of two different SM waveguides *A* and *B* are *on-diagonal* quasiperiodic due to the Fibonacci distributions of propagation constants *β*_A_ and *β*_B_ (see some description of on- and off-diagonal disordered arrays of waveguides in [21]). Notice that, the coupling coefficients between the nearest waveguides are the functions of the overlapping between the modes and the propagation constants of these waveguides^39^. Consequently, the coupling coefficients in our new FMCRFs also have quasiperiodic – or deterministically disordered distribution. Therefore, our FMCRFs provide platforms having *both on- and off-diagonal* deterministic disorders for realizing LQWs deterministically (e.g., both propagation constants and coupling coefficients are quasiperiodic).

## Quantum Walks in Periodic and Quasiperiodic Multicore Ring Fibers

In this section, first we will present the simulation of QWs and LQWs in MCRF and FMCRF, respectively. As mentioned in the Introduction, in this work we restricted ourselves to the problem of single-photon QWs. In general, simulations of single photon QWs in *irregular* arrays of waveguides are extremely difficult and numerical solutions are necessary. Especially if the propagation loss is included, solving numerically the Linblad equation would require large resources including computing time and memory^40,41^. However, we can make use of the very important fact that single photons QWs evolution are not different with propagation of coherent light; the distribution of light intensity represents the probability of photon detected at any position^1–3,20,42^. In fact, several important experiments of single photons QWs have been performed with coherent laser sources^1,3,20^. Theoretically, the description of a coherent light propagation in photonic lattices is completely analogous to the quantum description of the evolution of single photons QWs on the lattices^2,3,20,42^. As expected, experimental demonstrations of single photons QWs in photonic lattices are in very good agreement with the theoretical results in regular lattices^2,3^. Therefore, we can take advantages of the beam propagation method (BPM), one of the most effective simulation methods of light propagation to simulate the problem of single photons QWs in photonic lattices. We have developed our own Matlab program to simulate single photons QWs in periodic and quasiperiodic arrays of waveguides^37,38^, and MCRFs in this works. Note that BPM has been originally developed for decades^43,44^ and commercial software is also available. Our BPM programs have been successfully applied to simulate and design multimode cladding-pumped Er-doped fiber amplifiers^45^, Yb-doped multicore fiber lasers for the coherent Ising machine^46,47^, and also for single-photon quantum QWs in regular and irregular arrays of waveguides^37,38^. Details of the BPM method are presented generally in Refs. ^43,44^, its applications for simulating QWs in photonic lattices^37,38^ and the simulation results of QWs in MCRF and FMCRF are presented as follows.

**Figure 3:**
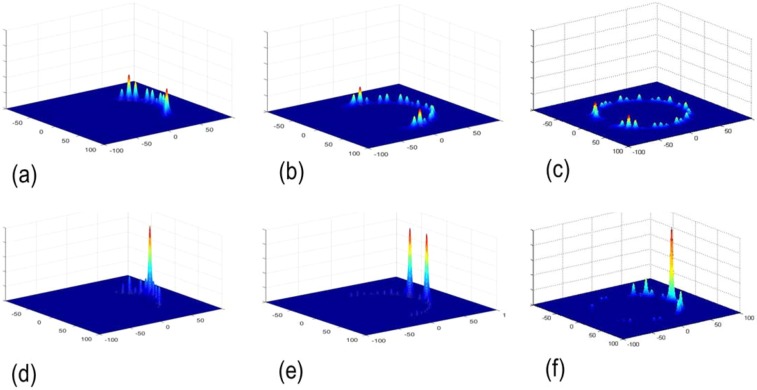
Probability distribution of photons in quantum walks: (**a**) regular MCRF with 13 cores, (**b**) regular MCRF with 23 cores and (**c**) regular MCRF with 39 cores, (**d**) FMCRF4 with 13 cores, (**e**) FMCRF5 with 23 cores and (**f**) FMCRF6 with 39 cores. Signal wavelength λ = 1.550 μm.

The simulations in Fig. 3 show the probability distribution of QWs in periodic MCRF with photons spread across the lattice by coupling from one waveguide to its neighbors in a pattern characterized by two strong “ballistic” lobes as in a typically normal QWs on a line. Meanwhile, the results for FMCRFs are structurally different: LQWs are clearly shown in quasiperiodic Fibonacci multicore fibers FMCRFs. Furthermore, symmetrical distributions of LQWs in FMCRFs can be achieved due to the symmetry of the quasi-periodic ring of cores in FMCRFs.

**Figure 4:**
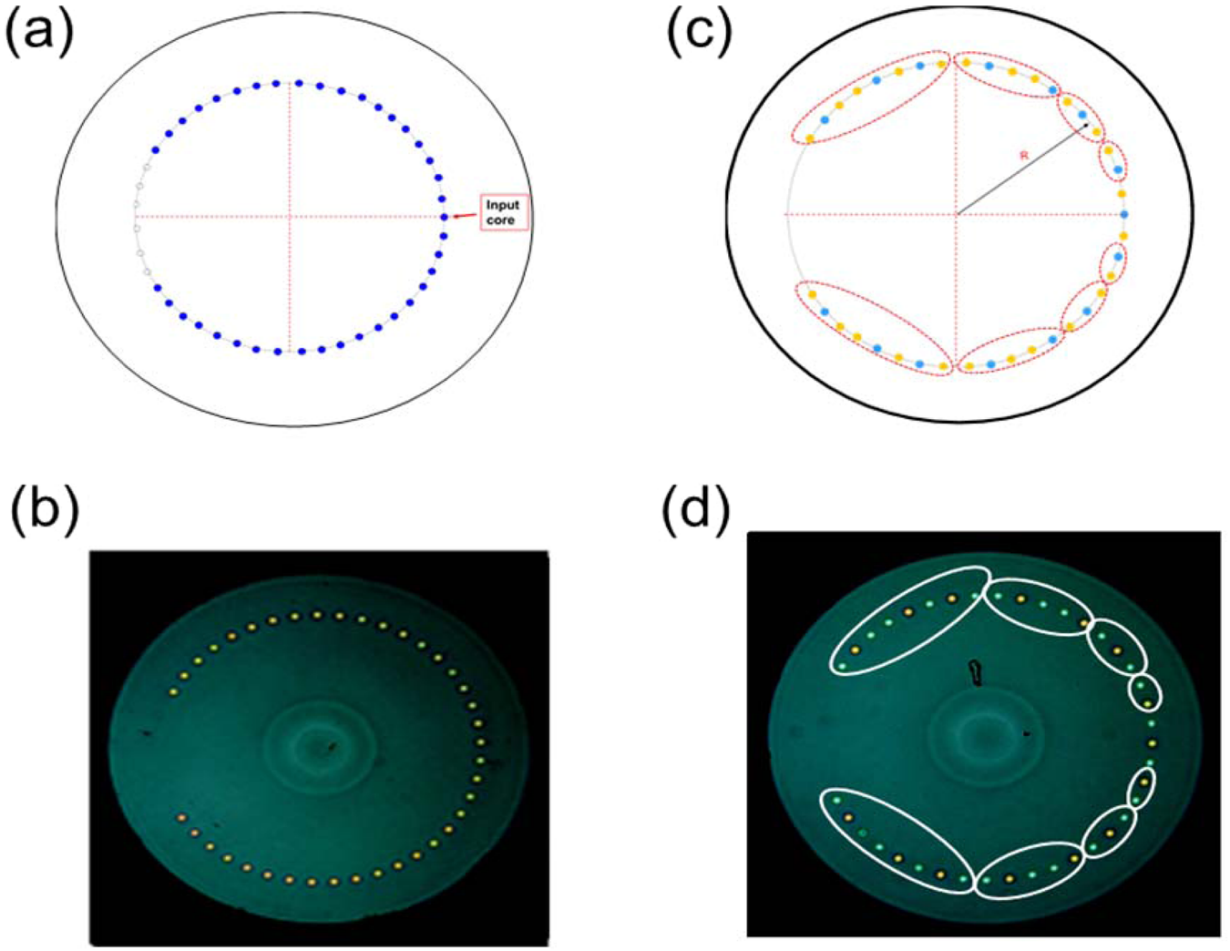
(**a**) Schematic of multicore fibers MCRF of 39 cores that are regular-spacing in a ring of radius *R*_1_. All cores have same index difference Δn and core size *a* and are single mode, (**b**) Image of cross section of MCRF fabricated with 39 cores. (**c**) Schematic of Fibonacci multicore ring fiber FMCRF of 39 cores that are regular-spacing in a ring of radius *R*. There are two different SM waveguides *A* (blue) and *B* (orange) having same core diameter *a* but difference Δ*n*_A_ = n_coreA_ − n_clad_ and Δ*n*_B_ = n_coreB_ − n_clad_. The circles around groups of cores are for different Fibonacci elements defined in Eq. (2). (**d**) Image of cross section of FMCRF fabricated with 39 cores. All parameters of the two fabricated fibers are detailed in the main text.

In order to demonstrate QWs and LQWs in periodic and quasiperiodic Fibonacci MCRFs (or FMCRFs), we have designed and fabricated this new type of fiber with ring of cores, both periodic and quasiperiodic ring of cores. Design and images of MCRF and FMCRF are shown in Fig. 4(a–d), respectively. We have performed experiments to realize single photon QWs in those fibers. Photon distribution of QWs in the MCRF and FMCRF are presented later in this section.

The two fabricated fibers, one is a periodic core ring - MCRF with 39 identical SM waveguides having core diameter *a* = 4.5 μm, index difference Δ*n* = *n*_core_ − *n*_clad_ = 0.0035; core-ring radius *R*_1_ = 120 μm, and clad diameter *R*_2_ = 160 μm. The quasi-periodic FMCRF is 6^th^ Fibonacci order that has R_1_ and R_2_ are the same as in MCRF, but its 39 cores are composed of two different SM waveguides *A* and *B* with the same core-diameter *a* = 4.5 μm, and different index differences Δ*n*_A_ = 0.0045 and Δ*n*_B_ = 0.0035. Note that, *A*- and *B*-waveguides are depicted by blue and yellow solid circles in Fig. 2 above. Our characterizations show the variation of core diameters in FMCRF is about +/−3% which is larger than +/−2% in MCRF which can be attributed to the different materials used in cores *A* and *B*-waveguides of FMCRF.

The MCRF and FMCRF both with 39 single mode cores were characterized using a cross-polarization microscopy. The microscopy system is a Nikon, high magnification optical microscope with error of ±0.05 μm. The average core diameter measured is ~4.45 μm and ~4.60μm for MCRF and FMCRF, respectively. The average distance from center-to-center of neighboring cores is ~16.89 μm and ~16.80 μm for MCRF and FMCRF, respectively. The index observed from crossed-polarization indicate that the CRF have identical refractive index for all of cores, whereas the FCRF have different core refractive indices grouped as described in previous sections. The core ring radius is approximately ~120 μm and fiber radius is approximately ~158 μm.

**Figure 5:**
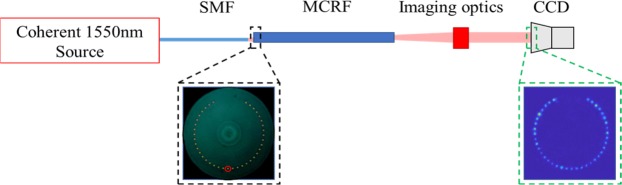
Experimental set up. A coherent 1550 nm laser output is coupled into a single mode fiber (SMF) attached onto an XYZ stage. Light from SMF is then butt-coupled to the MCRF at the central core indicated by the black inset with red circle. The output from MCRF is imaged onto a CCD camera, green inset. The SMF fiber is chosen such that the MFD matches that of the MCRF.

Demonstration of quantum walks in MCRF and FMCRF were conducted with a stripped fiber, at approximately 4-cm. The fiber is placed on a v-groove in an imaging system shown in Fig. 5. A tunable source from 1510–1590 nm laser illuminates the MCRF/FMCRF. The steps taken to identify the central core and the measured quantum walk distribution is as follows: First, we Illuminate subsections of the fiber of interest (FOI) such that cores are illuminated. As an example, the image of the illuminated MCRF is shown in Fig. 1c. Next, we combine illumination images to identify the position of each cores using the Matlab/Labview algorithms to determine the diameter and position of each core of the FOI. From that characterization the position of the central core is determined for the input signals in the quantum walks experiments. Once we know the position of the input core, we launch a coherent beam of light into it by butt-coupling with a SM fiber that is mode-matched to the input core of MCRF and FMCRF. The image of output facet is captured and output signal from all cores are measured. We repeat those steps for wavelength sweep from 1530–1559 nm to account for fiber length variations. Matlab codes are written to calculate the total intensity for each core.

**Figure 6:**
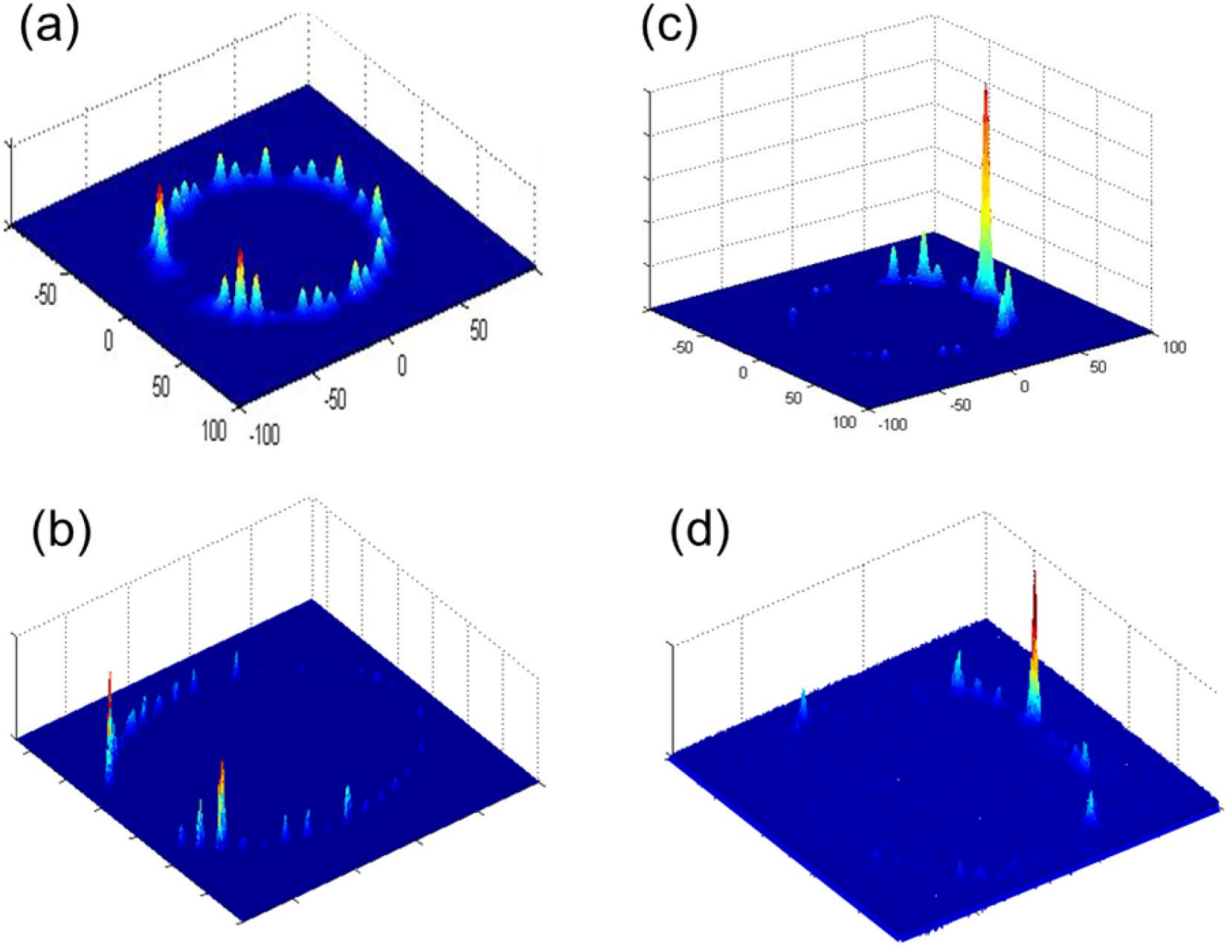
(**a**) Calculated probability photon distribution of quantum walks in the MCRF, and (**b**) experimental data of photon distribution at ~4.1 cm of MCRF. Both simulation and experimental results show the typical feature of QWs with two strong lobes at the end of walking length. (**c**) calculated probability photon distribution of quantum walks in the FMCRF, and (**d**) experimental data of the photon distribution at ~4.15 cm of FMCRF. The simulation and experimental results show strong localization at the center core. Details of the fibers and experiments are presented in the main text.

We show in Fig. 6 both simulation and experimental results of single photons QWs in MCRF and FMCRF. Figure 6a,c are the simulations and Figs. 6b,d are experiment results of photon distribution of QWs in MCRF and FMCRF, respectively.

The results in Fig. 6(a,b) show probability distributions of single photons QWs in periodic MCRF spread across the lattice by coupling from one waveguide to its neighbors in a pattern characterized by two strong “ballistic” lobes. Note that the results are measured at ~4.1 cm of fiber length, and typical characteristics of QWs on a line are clearly shown. The experimental data is in very good agreement with simulation results, both qualitative and quantitative. It is worth to mention here that experiments of QWs in [2, 3] with lattice of identical waveguides fabricated on an AlGaAs substrate waveguide is about in 8 mm long, in laser-writing photonic lattices^5^ is less than 1 cm. The QWs behavior can only be preserved at around 9.81 mm of the laser-written waveguides lattice, and it is completely distorted if walking longer distances. Our experimental results show QWs characteristics are clearly preserved in ~ 4 cm length of MCRF indicate that both losses and imperfection in our MCRF are very good for QWs experiments.

In Fig. 6(c,d) we show the results of QWs in FMCRF at ~4.15 cm. As stated earlier, this quasiperiodic fiber – the FMCRF is composed of two different waveguides of different materials. As a result, the FMCRF has variation of core diameters +/−3% which is larger than in MCRF. Even with that, the strong localization in the center core as predicted in the simulation is clearly preserved in Fig. 6d. However, the imperfection of the core sizes and core-to-core distances is the reason for slight distortion of QWs behavior resulting in an unsymmetrical distribution of photons observed in the experiment. Meanwhile, modeling results in the ideal conditions predict a symmetrical distribution of QWs as shown in Fig. 6c. It is clear that the experimental results are in a very good agreement with the simulations, except for small discrepancies due to unavoidable imperfections.

The simulation and experiment results in Fig. 6 show two typically different QWs in periodic (ordered) and quasiperiodic (deterministically disordered) core rings. In the ordered system - the MCRF, the expected distribution for typical QWs on a line has been observed experimentally with two strong “ballistic” lobes. On the hand, the quasiperiodic or deterministically disordered Fibonacci MCRF shows localized QWs as predicted by the simulations. Note that our experimental results of QWs and LQWs in MCRF and FMCRF, respectively can be further improved as several factors such as coupling misalignment, surface roughness, reflection by air/cladding interface can be reduced significantly from the current set up. Misalignment, roughness, air/cladding interfaces cause distortion and unwanted localization and interferences in the fiber. These issues can be resolved using index matching oil to reduce reflections at the interface between cladding and air. To achieve a short-length fiber with minimal roughness to the end faces from poor cleaving or from unwanted back-reflection from flat end faces, we fabricated a housing unit for the fiber made of angled-ferrules and/or canes filled completely or in part with index-matching oil and high-refractive index adhesive. The ferrules are polished at an angle or flat depending on tolerance for back-reflection. Optimization of the fiber-drawing process will improve the variation of core sizes in the fibers.

It is clear from simulation and experimental results in Fig. 6, LQWs in quasiperiodic Fibonacci MCRF are predictable and controllable due to the deterministic disorder nature of the system. Moreover, symmetrically distributed LQWs can be realized not only in the Fibonacci MCRF as demonstrated in this work, but also in the new proposed class of QPLs constructed with Fibonacci and other quasiperiodic sequences^37,38^. These are unique features of LQWs in QPLs and potentially important for several quantum applications. For example, Chandrashekar and Busch have recently proposed employing symmetrically distributed LQWs for quantum memory applications^24^. In their proposal, temporally disordered operations in spatially ordered systems are used to realize symmetrically distributed LQWs. However, it is very challenging to implement the approach because multiple quantum coins operations are very difficult in practice^24,48^.

It is important to note that localizations of light in QPLs have recently been investigated theoretically and experimentally^49,50^. These QPLs are arrays of identical waveguides constructed with quasiperiodic Fibonacci sequence in distances. In general, those QPLs have the same definitions of Fibonacci elements as in Eq. (1) of this work. However, in these works *A* and *B* were defined as the two fundamental distances 1 (unit) and golden ratio τ = 1.618 between the nearest waveguides, respectively^49,50^. Because all waveguides in these Fibonacci elements are identical, quasiperiodic properties were attributed to the golden ratio spacing of Fibonacci sequence^49,50^. These QPLs are constructed with Fibonacci spacing sequence (FSS) or FSS-QPLs for short. In contrast, quasiperiodic properties of our new class of QPLs that have recently been proposed^37,38^ and are demonstrated in this work are determined by quasiperiodic sequences such as Fibonacci, Thue-Morse etc. of two different waveguides *A* and *B*. As pointed out in our previous work^38^ it is not necessary to have spacing with golden ratio for realizing LQWs in FSS-QPLs. We have numerically demonstrated that the quasiperiodic Fibonacci sequence itself is responsible for deterministic disorders in FSS-QPLs even with different spacings^38^. Finally, it is worth to stress here that the FSS-QPLs could be classified as off-diagonal deterministic disordered QPLs. These FSS-QPLs are composed of identical waveguides (same propagation constants) but they have quasiperiodic distributions of coupling coefficients (off-diagonal) due to quasiperiodic spacings. Meanwhile our proposed QPLs, and the Fibonacci MCRFs in particular, are constructed with Fibonacci sequences of two different waveguides generating deterministic disorders of both propagation constants (on-diagonal) and coupling coefficients (off-diagonal).

## Discussion and Conclusion

First, it is worth noting that although single-photon QWs do not explore the true quantum nature of photons, the effect and its demonstration are very important not only for understanding the QWs effect itself, but also very meaningful in research and developing protocols of quantum applications^16,51–55^. More importantly, there have been important quantum applications that make use of single-photon quantum walks, in particular in quantum communication and quantum information^51^. In quantum communication, the two photons of an entangled pair do not necessarily propagate together in the same devices and processing singlephotons is critical for quantum communication. Several well-known quantum system protocols, for example the quantum key distribution (QKD) protocol BB84^52^, and KLM protocol of linear optical quantum computing (LOQC) demand single photons^53^. The QKD BB84 demand single photons traveling over a channel, first proposed by Bennett and Brassard in 1984^52^ and discussed with emphasizing the important of single photons in quantum applications^51^. Significantly, in 2000 Knill, Laflamme and Milburn proposed a new protocol (KLM protocol) aiming at creation of universal quantum computers using linear optical elements, single photon sources and photon detectors^53^. Since then, the KLM protocol have inspired great efforts of investigation of QWs of photons in photonic lattices^1–5,37,38,42^. In addition to that, an efficient idea for quantum repeaters based on single-photon sources has been first proposed by Sangouard *et al*.^54^, and later a protocol based on that idea has been implemented with atomic ensembles and a single photon source with repetition rate of 10 MHz^55^. Regarding to quantum walk phenomena in photonic lattices, the authors of Ref. ^42^ present a very good review both on single-photons and multiple-photon QWs. As for potential applications of single photons QWs in Fibonacci MCRFs, or more general – quasiperiodic photonic lattices, one can use two separate structures for storing and retrieving each photons of entangled pairs in quantum memory applications^37,38^. Secondly, given the importance of single photons QWs, we want to stress here that the results of multi-photon quantum walks would explore true quantum nature of photons and therefore can be directly used in many important applications in quantum technology. Therefore, theoretical and experimental investigation of multi-photon QWs in general, particular in our periodic and quasiperiodic Fibonacci MCRFs have been undergoing in order to gain meaningful results for different quantum applications.

In conclusion, for the first time QWs and LQWs have been demonstrated in periodic and quasiperiodic multicore ring fibers, respectively. The new multicore-ring fibers (MCRF) provide a new scalable platform for experiments of QWs and other quantum effects that require extremely low insertion and large numbers of waveguides. The core structure in quasiperiodic FMCRF is constructed with Fibonacci sequence provides a new class of QPLs that possess both on- and off-diagonal deterministic disorders. Although the structures of the quasi-periodic Fibonacci lattices are straightforward to fabricate, the outcome results of LQWs are predictable and controllable, in contrast with LQWs in randomly disordered systems. The new class of QPL thus provide platforms for realizing LQWs and for investigation of quantum effects with both on- and off-diagonal deterministic disorders. Furthermore, the new proposed *j*^*th*^-order QPLs are constructed as an orderly sequence chain of all elements up to *j*^*th*^-order instead of individual *j*^*th*^-order Fibonacci elements as in refs. ^49,50^, which are convenient to make QPLs symmetric that could be of benefit for some special applications^24,48^. Furthermore, the rule of construction for symmetrical QPLs can be applied with other quasiperiodic sequences such as Thue-Morse^48,56^, and Rudin–Shapiro^48^ sequences. It is worth noting that high-dimensional QWs in multimode cores have recently demonstrated^57^ revealing some new and interesting results. Using wavefront shaping, the authors can control the two-photon states in multimode fiber core that support ~380 modes^57^. Our results in this work are with single-mode cores at photons wavelength 1.55 μm, therefore the results are conventional 1D QWs on a line. It is interesting to point out that the cores of our MCRFs both periodic and quasiperiodic ones are multimode in shorter wavelengths, for example 850 nm. Therefore, our MCRFs can be used as new platforms for investigating multi-dimensional QWs using 850 nm light. Finally, we would like to stress that although some applications would benefit from small-scale structures as our current MCRF scale, or even smaller structures like on-chip scales, we would like to stress that other applications would require much longer-distance lengths of MCRFs. In such situations, special deployments conditions and efforts must be considered for maintaining phase and polarization stability in multicore fibers. Several methods have been recently proposed and developed for auto-compensating phase and polarization in quantum cryptography in multicore optical fibers^58–60^. We believe that modifications of these methods can be applied in many different quantum problems including the one of QWs in our MCRFs.

## Methods

Fabrication of the multicore ring fibers. In order to demonstrate QWs and LQWs in periodic and quasi-periodic MCRFs, we have designed and fabricated this new type of fiber with ring of cores, both periodic and quasi-periodic ring of cores. The fabrication method of MCRFs and FMCRFs includes fabricating disc-shaped segments for cladding glass by polishing flats and drilling bores in the direction orthogonal to the flats stacking a number of disc-shaped segments to form a combined cladding glass part, so that bores of all the disks match, and inserting continuous core cane segments into the bores sealing flats of the disc segments to each other, sealing bores to the core canes, and drawing multi-core preform into a multi-core fiber. This method provides a robust and cost-effective process for manufacturing of precision multi-core fiber. Core-drilling provides precision and robustness. Stack-sealing enables the use of short and bulky precision drilled parts of cladding glass for constructing a multi-core fiber preform. Such preform may also include solid end pieces of glass for improved utilization of precision drilled parts in the fiber draw. Vacuum is used is used to hold the stack together in the furnace while it is being sealed. Sealing is achieved in all directions simultaneously. The use of temperatures above glass softening point enables sealing surfaces with just fine grind finish.

Modeling and simulation. As single photons QWs do not behave any differently from classically coherent wave propagation, and the distribution of light intensity corresponds to the probability distribution of photons. Therefore, we can use the beam propagation method (BPM)^43–45^, one of the most effective methods of light propagation simulation for simulating single photons QWs in MCRFs. We start from the wave equation in paraxial approximation for the slowly varying electric field *E*(*x,y,z*) propagating in *z*-direction

3 $$\frac{d}{dz}E(x,y,z)=(\hat{D}+\hat{V})E(x,y,z).$$

The diffraction $\hat{D}$ and inhomogeneous operators $\hat{V}$ are given by

4 $$\hat{D}=\frac{i}{2k}\left(\frac{{\partial }^{2}}{\partial {x}^{2}}+\frac{{\partial }^{2}}{\partial {y}^{2}}\right),\,\hat{V}=\{ik\Delta n(x,y,z)-\alpha (x,y,z)\}.$$

In Eq. (4) $k={n}_{0}{k}_{0}=2\pi {n}_{0}/\lambda$ where *n*_0_ is the background or reference refractive index and λ is the free-space wavelength, $\Delta {\rm{n}}=n(x,y,z)-{n}_{0}$ is the refractive-index profile relative to the reference refractive index, and α is the power absorption/loss of the waveguide. A small propagation step is implemented using the following approximation:

5 $$E(x,y,z+\Delta z)={e}^{(\hat{D}+\hat{V})\Delta z}E(x,y,z)\approx {e}^{\frac{\hat{D}\Delta z}{2}}{e}^{\hat{V}\Delta z}{e}^{\frac{\hat{D}\Delta z}{2}}E(x,y,z).$$

The BPM solution, e.g., Eq. (8) can be solved very effectively by fast Fourier transformation (FFT) algorithm^43–45^. In simulations, the input field at input core (coordinates *x*_0_, *y*_0_) has the form

6 $$E(x,y,z=0)={A}_{0}\exp \left(-\frac{{(x-{x}_{0})}^{2}}{{\omega }_{0}^{2}}-\frac{{(y-{y}_{0})}^{2}}{{\omega }_{0}^{2}}\right).$$

here, ${\omega }_{0x}={\omega }_{0x}={\omega }_{0}$ is spot-size of the input beam with amplitude *A*_0_.

It is worth to stress again that the modeling method described above is valid only for problems of *single photon QWs*. Modeling indistinguishable or entangled photons QWs is more complicated, aiming to explore the non-classical nature of photons. Even in the simplest case of two indistinguishable photons injected into the photonic lattice, non-classical nature of light can be observed theoretically and experimentally^1,3^. The results show that when both indistinguishable photons are coupled to a single waveguide at the center of the lattice, the correlation matrix is a simple product of two single-photon distributions, thus showing no quantum interference. However, when the two photons are coupled to two adjacent waveguides the two photons exhibit bunching and will emerge from the same side of the lattice, which can be explained as a result of a generalized Hong-Ou-Mandel interference^1,3,42^. Significantly, the results show that the photon density ${n}_{q}= < {a}_{q}^{\dagger }{a}_{q}\, >$ is directly determined by the classical light intensity and carries no quantum signature, here ${a}_{q}^{\dagger }\,{\rm{and}}\,{a}_{q}$ are creator and annihilator operator of photon, respectively. Furthermore, the results also show this behavior hold for not only separable but also path- entangled input states^3,42^. This fact indicates that our modeling and simulation method in this work is still relevant as the method could provide important information of the photon density in problems of multiphoton QWs. Again, quantum effects can be observed in the correlation function ${\Gamma }_{q,r}= < {a}_{q}^{\dagger }{a}_{r}^{\dagger }{a}_{q}{a}_{q}\, >$. The evolution of correlated or entangled particles in randomly disordered lattices was investigated in off-diagonal disordered lattices^61^. The results show that while the particle density *n*_*q*_ evolves following the single-particle dynamics and exhibits Anderson localization, the two-particle correlation ${\Gamma }_{q,r}= < {a}_{q}^{\dagger }{a}_{r}^{\dagger }{a}_{q}{a}_{q}\, >$ develops unique features that depend on the quantum statistics of the particles and their initial separation^61^. Investigation of multiphoton QWs in MCRFs, and Fibonacci MCRFs would reveal new features of photons in periodic and quasiperiodic photonic lattices.

## Data Availability

The data that support the plots within this paper are available from the corresponding author on reasonable request.
